# Progress in Surgery

**Published:** 1896-10-10

**Authors:** 


### Progress in Surgery.

### GENITO-URINARY SURGERY.

**Rupture of the Bladder and Urethra.**—A case of successful suture of a ruptured bladder in a female is recorded by Heaton, of Birmingham.[1] It is the only successful case in a woman on record. There were fairly typical symptoms of ruptured bladder, and the diagnosis was confirmed by the passage of a large silver catheter, which could be passed its whole length without meeting with any resistance, and could then be felt high up in the abdomen beneath the anterior abdominal wall. No urine escaped until the catheter was withdrawn within three inches of its end, when four ounces of bloody urine escaped. A rent three inches long was discovered on the upper and posterior aspect of the bladder. It was sutured with the finest silk, and the security of the junction tested by injecting the bladder The abdominal cavity was washed out with warm water. Heaton points out that the shock in some of the recorded cases of ruptured bladder has been very slight, and patients have been known to walk to hospital and go away again with a rupture of the bladder undiscovered. The author has collected the records of thirty-seven cases of ruptured bladder in which the abdomen was opened. In six cases no suture was employed, and three recovered. In two of these three successful cases a tampon of iodoform gauze was placed in the abdomen behind the bladder. Out of thirty-one cases in which the rent was sutured, twelve cases were successful and nineteen unsuccessful. In eleven cases death was due to peritonitis, either previously in existence or following the operation. In four of the cases of peritonitis the suturing had not been secure enough, and the sutures had given way in each case at the posterior angle of the wound, where it is always the most difficult place to apply them. In seven cases the peritonitis preceded the operation. In five cases death was due to shock. In only two of the successful cases was peritonitis present at the time of operation. The necessity of early operation is therefore apparent. In the "Progress in Surgery" in The Hospital for April 6th, 1895, attention was called to recorded cases of rupture of the bladder, in which the presence of urine which had extravasated into the peritoneal cavity for some days, did not set up any peritonitis, and to experiments on animals in which a similar result was obtained. It is supposed that patients die from urinary absorption. Heaton refers to this fact. The methods usually employed to determine the presence of a rupture in the bladder wall are mentioned, and attention is called to one employed by Mr. Walsham in a recent case,[2] to which we referred some time ago, in which Mr. Walsham discovered the rent by injecting air into the bladder with the hand ball-pump used for the ether-freeing microtome. The air passed through the rent, and caused abnormal resonance in the abdomen, but serious collapse came on apparently as a result of the injection of air into the peritoneum. This collapse was relieved as soon as the abdomen was opened, so that Mr. Walsham advised that this method should only be employed when the surgeon is quite ready to operate. If, after the employment of other means, there is still uncertainty as to the existence of rupture Mr. Heaton recommends that an exploratory incision should be made through the abdominal wall just above the pubes, and if no extra peritoneal rupture is discovered, the peritoneal cavity should be opened and the upper and posterior aspect of the bladder explored.

Dr. Weir, of New York, advocates[3] immediate suture in cases of ruptured urethra, combined with drainage of the bladder by the suprapubic route. He thinks suturing the torn urethra will lessen the risks of serious stricture, but will not be certain to prevent the formation of a stricture. The retention of a catheter in the bladder, after suturing the urethra, has the disadvantages that it may set up cystitis and may allow urine to flow along the urethra by its side. To avoid these the author advises that after the urethra is sutured no catheter should be retained, but the bladder drained from above the pubes. If there is difficulty in finding the posterior end of the torn urethra he suggests that by directing a fine douche of hot water on the perineal wound for at least five or ten minutes with firm sponging, the blood will be washed away and the torn end possibly recognised. If not, he thinks the bladder should be opened above the pubes, and a sound passed from the interior along the urethra as a guide to its proximal part, and the bladder opening used for subsequent drainage Although it is not easy to reach the bladder in the empty condition without risk of wounding the peritoneum, Weir thinks, by keeping very close to the pubes it may be safely done. Judging from the mortality statistics of ruptured urethra, the retention of a catheter without perineal incision is not advisable, the cases treated by perineal incision having a mortality of only half those in which a catheter was retained and no incision in the perineum made. In twenty-nine cases of immediate suture there were no deaths, and in nearly all these the bladder was drained by a retained catheter; only in a few was the bladder drained by a suprapubic opening; nearly always the perineal wound, after the torn urethra had been sutured, was left partly or wholly open. In a few cases in which there was only slight blood extravasation and no apparent urinary infiltration, the perineal wound was closed. Weir does not recommend any attempt to close the perineal wound, but only advises that it should be approximated sufficiently to support the newly-joined urethra. In most of the successful cases cat-gut was used as suture, and the mucous membrane was not included in the stitches. Weir uses silk, passes it through all the coats, and leaves the ends long, so that they can be pulled away. In nineteen cases, six were free from signs of stricture seven to eighteen months after the operation. The author considers that in an injury of the perineum with painful or difficult urination, accompanied by a free flow of blood, with or without perineal or scrotal swelling, a free perineal incision should be made and the urethra exposed; and if this canal be ruptured it should be sutured, and the bladder drained, preferably by a suprapubic opening, and by the use of Cathcart's or Dawbarn's apparatus for siphon drainage. Reference is made to a case[4] in which the urethra was ruptured by a bicycle accident. The bicycle rider plunged into a ditch two feet deep, and the perineum received extensive damage. Weir records two cases of his own of ruptured urethra. In both cases the torn urethra was sutured, and in one the bladder was drained from above the pubes, and in the other by the retention of a catheter. In one case, although the urethra was found completely torn across in its bulbous portion, and there was a separation of the ends of the canal for three-fourths of an inch, yet urine was passed in small quantities for three days, and no extravasation of urine seems to have occurred. The patient was admitted to hospital on the fourth day with an enormously-distended bladder. Dr. MacBurney[5] considers suturing the torn urethra unnecessary, the results, after simple perineal incision and catheterisation, he thinks are so good; but the tendency to stricture Dr. Weir thinks considerably lessened by suturing, and he points out that the mortality has been less in cases in which the urethra has been sutured than in those in which the surgeon has been content with simply making a perineal incision.

At a meeting of the American Association of Genito-Urinary Surgeons, Cabot, of Boston, reported[6] five cases of ruptured urethra treated by external urethrotomy and suture. He refers to the intractable nature of traumatic stricture, which in some cases is so marked that even with the frequent passage of an instrument it is very difficult to keep the stricture dilated. In two of the cases in which he sutured the urethra no stricture was discovered several years after any dilating instrument had been passed. He thinks that "in every case of ruptured urethra immediate perineal section, with suture of the urethra, should be practiced," a proceeding which he considers greatly lessens the dangers of urinary extravasation, and also prevents the formation of tight, intractable strictures. The sutures employed were of catgut, and included the cavernous and muscular tissue only. Dr. W. White, of Philadelphia, agreed with the author. He thought it doubtful whether it was needful to suture very slight cases of rupture in which there was practically no symptoms except a little hæmorrhage and some perineal swelling. Mr. Herbert Page[7] describes a case of rupture of the membranous part of the urethra in a child from fracture of the pelvis, in which blood-clot and urine collected in the prevesical space (Caveum Retzii), and led to the belief that the clot had been intravesical. The boy was run over, and when admitted there was slight bleeding from the urethra, and a catheter, which was believed to have entered the bladder, withdrew only blood. This bleeding was supposed to be of renal origin, and no injury to the bladder was suspected. A large hypogastric swelling formed, and as no catheter could be introduced into the bladder (which the hypogastric swelling was supposed to be), the swelling was punctured above the pubes, and blood-clot evacuated. At the *post-mortem* examination the bladder and kidneys were found uninjured. Reference is given to several other cases, illustrating the surgery of the prevesical space.

[1] Annals of Surgery, June, 1896, p. 676. [2] Trans. Medico-Chirurgical Society, Vol. lxxviii. [3] New York Med. Record, May 9th, 1896, p. 651. [4] Gaz. des Hôp., Jan. 2nd, 1896. [5] New York Med. Record, May 9th, 1896, p. 662. [6] Journal of Cutaneous and Genito-Urinary Diseases, July, 1896, p. 269. [7] Brit. Med. Journal, July 18th, 1896, p. 113.

### SURGERY OF THE NERVES.

*(Continued from page 11.)*

**Surgical Treatment of Trifacial Neuralgia.**—Among the numerous surgeons who have attempted, by intra-cranial operations, to combat this—perhaps the most distressing of all nervous diseases—Krause[2] is pre-eminent. He recommends the removal of the Gasserian ganglion, in addition to section of the divisions of the fifth nerve, having had experience of recurrence of pain soon after the latter operation alone. The physiological importance of the ganglion has been exaggerated, any trophic disturbances which follow its destruction being but slight and transient. A flap of scalp and bone is raised in the zygomatic region, the dura separated from the bone, and with the brain lifted up on a flat director till the ganglion is brought into view, the second and third divisions of the nerve are divided at their foramina of exit, and the ganglion seized with forceps and forcibly dragged out along with the nerve trunk. The first division is not dissected out on account of its proximity to the cavernous sinus, but is torn across. The two great risks of the operation are hæmorrhage from the dura and sinuses, and compression of the brain. The former is met by limiting the area of separation and the use of gauze tampons; and the latter by slow, gradual, and moderate raising of the brain from the base of the skull. The ages of Krause's patients ranged from thirty-six to to seventy-two. He had no case of recurrence after removal of the ganglion. The oldest of his cases have been operated upon two and a quarter and two years respectively.

Tiffany[3] has had cases in which the pain remained after the removal of the ganglion and nerve, and other American surgeons have had similar experience. One of them was explained by the finding *post-mortem* of a neuroma on the stump in the foramen rotundum. The only physiological effects have been anæsthesia of the conjunctiva, nasal and buccal mucous membrane, and paralysis of the muscles of mastication when the motor root has been destroyed. Keen[3] has had trouble with the cornea in three cases, but it was never severe. Tiffany estimates that about 100 operations have been published by forty-five operators, of whom twenty-four have operated on one case only. The mortality is about 10 per cent., but will probably improve when operator have gained experience in the procedure.

Other operative measures have been tried. Morton[3], of Philadelphia, had a case in which in less than two years after removal of Meckel's ganglion pain recurred with great violence. The common carotid was ligatured, and permanent relief followed. Park[4] has successfully ligatured the carotid in two cases. It is to be borne in mind that the mortality of this operation carried out in a healthy blood vessel is 5 per cent.

**Thiersch's Method.**—This consists in slow extraction of the central end of the nerve trunk and of the peripheral end at the point of its emergence from the osseous canal. Angerer, of Munich,[5] gives the results of twenty-five operations for trifacial neuralgia by this method. In all the cases save four the disease dated back beyond a year. In seventeen cases the results were entirely satisfactory; in seven there was recurrence with uncertain results. In seven cases the pain has been absent for four years after operation. The results Angerer thinks much superior to simple resection of the nerve at the point of exit. The avulsion should be performed very slowly, and should be applied to all the branches of the nerve, although only one be affected with pain. In cases of relapse Krause's operation is indicated. Helferich[5] emphasises the importance of thorough avulsion of the central end of the nerve. He has operated on twelve cases, and Karewski[5] on nine, but the ultimate results are not stated.

**Nerve Suturing and Nerve Loosening.**—Wölfler[6] reports on ten cases operated on for paralysis of peripheral nerves, as shown in following table:—

A. Suture of Nerves.

I. Direct union.

*a.* Primary.

1. Facial nerve after injury. Complete recovery.
2. Median and radial nerves.

*b.* Secondary.

3. Median N. Complete recovery.
4. Radial N. Complicating fracture. Recovery.
5. Cervical Plexus V. and VI. Recovery.

II. Indirect union.

6. Radial N. Formation of loop. Result ?
7. Peroneal N. Nerve grafting. Result ?

B. Loosening of Nerves.

8. Peroneal N. After Osteotomy. Recovery. (7 years' duration.)
9. Radial N. Fracture. Recovery.
10. Radial N. Exostosis. Recovery.

The writer advises an operation in all cases whenever paralysis is established, no matter how soon or how long after injury this takes place. The reunited nerve must be placed in as natural physiological surroundings as possible to prevent its being implicated in the cicatrix, or being kinked as tissues contract. Primary union is essential. The commonest indication for loosening nerves is where the nerve has become implicated by adhesion in fractures of the upper arm. Conservative treatment may be employed, but has usually to be followed by operation.

Bradley[7] grafted a piece of the sciatic nerve of a dog into the musculo-spiral nerve of a trooper in the U.S. Army, who had sustained a fracture of the humerus, implicating the nerve. There was early evidence of returning sensibility, but the ultimate result is not reported.

**Section of the Cervical Sympathetic in Exophthalmos.**—Jaboulay[8] has found that section of the cervical sympathetic between the middle and superior ganglia in cases of exophthalmic goître, results in permanent disappearance of the exophthamos, with temporary amelioration of the other symptoms.

**Anatomy of Lenticular Ganglion.**—Fawcett[9] describes a dissection for the exposure of the lenticular ganglion and its branches. He exposes it by removing the outer wall of the orbit, lifting aside the external rectus muscle, and working down to the ganglion, which, when seen, is cleaned up by pouring over the parts some pure spirit, which causes the ganglion and its branches to stand out very prominently.

[2] Verhandlungen der deutschen Gesellschaft für Chirurgie, 1895. [3] N. Y. Med. Record, May 26. [4] N. Y. Med. Jour., May 26. [5] Med. Week, June 5, 1896. [6] Prager Med. Wochensh. 1895, No. 47. [7] Amer. Med. News, 1896. [8] Lyon Medical, 1896. [9] Lancet, April, 1896.

### DISEASES OF THE UPPER AIR PASSAGES.

**Adenoid Growths.**—Helme,[1] at the Soc. Franc. d'ot. et de Laryng., has advocated freshly-prepared bromide of ethyl as the most suitable anæsthetic. He states that after operating no dressings are to be used, especially no washing of any kind. Food should consist of iced milk and bouillon on the first day; eggs, puddings, and cooked fruits may be allowed the second day; and by the third day ordinary diet. Properly speaking, there is no recurrence of adenoids [a statement with which we cannot agree.—Ed.] Apparent recurrence is due to incomplete operation; true recurrence may occur in syphilitic tuberculous, or malignant tumours. As a rule, improvement is immediate and marked, but in strenuous cases it may be less so. In these one should carry out local treatment, consisting of painting the naso-pharynx with resorcin and glycerine, also general treatment (thermal, sea-air, &c.).

Lastly, there are the tuberculous adenoids. Of these there are two types: (1) Bacillary adenoids (Lermoyez), *i.e.*, where the bacilli are found inside the tissues—very rare, only one to seventy-five cases; (2) bacilliferous adenoids, *i.e.*, when the bacilli are found on the surface of the growths (Dieulofoy)—one to five cases. Garel[2] records two cases of hereditary syphilis simulating adenoid vegetations. The first case had been operated on by a colleague, and eight days later perforation of the palate was found. The second case was a young girl with the typical fauces of adenoids. M. Garel refused to operate on account of a serious cardiac lesion. Two months later the breaking down of a gumma caused perforation of the palate. Both cases rapidly recovered under iodide of potassium.

The dangers of this operation for adenoid growths should never be forgotten by any one operating. A point well illustrated in Lambert Lack's[3] experience of a case of postnasal adenoids under his care whose life was preserved by his having tracheotomy instruments at hand during the operation. Herbert Tilley[4] refers to a case of almost fatal asphyxia during operation for adenoid growths; the portion of the growth which had slipped into the glottis, however, was loosened by forcible pushing upwards of the larynx. He pointed out the advantage of having the patient's head hanging well down during the operation, so as to obviate the accident of a piece of removed growth or blood-clot falling into the larynx.

**Septal Deviations.**—J. N. Roe,[5] of Rochester, N.Y., refers these conditions to various causes, the two main predisposing causes being diathesis and racial characteristics. The principal diathetic influences are strumous, syphilitic, tuberculous, or brachitic diathesis. The influence of racial characteristics is shown by the greater prevalence of deviated septa among civilised than among savage races, and in the aquiline type of nose. The exciting causes may be internal or external. Internal causes: (*a*) Defective development, (*b*) diseases of the septum, (*c*) diseases of other portions of the nose. In discussing defective development, the author pointed out the fact that the frequency of deflections from this cause was due to the fact that in early life the vomer is composed of two laminæ, which are separated by a plate of fibro-cartilage, which is prolonged forward to form the cartilaginous portion of the septum. Ossification begins in each plate about the sixth or eighth week of fœtal life, but is not complete until after puberty. The coalescence of the laminæ takes place from behind forward, beginning about the third year. Bearing in mind the fact that the vomer is composed of two parallel laminæ which do not fully coalesce until after puberty, and in some cases do not coalesce at all, we can readily see the effect that would be occasioned by the slightest imperfect development of either of these plates. The fact that the laminæ coalesce from behind forward is the reason why we so rarely observe a deviation posteriorly, while the middle and anterior portions are so frequently deviated. But the unequal growth of the two plates may be influenced by disease itself.

Of the external exciting causes, the main one is traumatism. A. W. Watson[6] (Philadelphia) insists on the importance of treating septal deviation, firstly, by the removal of a portion of tissue in the general line of deviation. If the latter is horizontal, we must exsect an elliptical portion gradually convergent at both ends; if vertical, a wedge-shaped piece must be removed, its apex being above and its base lying near that of the septum, where, if necessary, it may be joined by a horizontal incision. Care must be taken to avoid cutting the mucous membrane of the sound side opposite the incision, as it helps to hold the edges in line, thus facilitating union and avoiding perforation. Incision must be made in the convex side of the deviation. Secondly, and of no less importance, the septum must be retained in position. Healing of the cartilage requires from three to four weeks. The best support is furnished by a flat ring-head pin, the latter being encased in rubber tubing. The pin should enter from the concave side of the septum, just back of its anterior edge and passed diagonally through to its other side, then across the vertical incision, if there is one, and then back again into the septum, until the head of the pin lies on the latter within the nostril. In this way, both nares are left free for breathing and cleansing. Padding of the pin-head with rubber prevents ulceration, and the pin may be worn for even as much as three weeks without any discomfort.

**Tuberculosis of the Nose.**—Wm. Hill[7] records a case of tuberculosis of the nose, which he remarks is a very rare disease, and is difficult to diagnose without a microscopical examination. Undoubtedly the patient was tuberculous, but the case to him presented many of the characteristics of malignant disease. There was pain in the nose, a tumour (with an ulcerating granular surface) of the inferior turbinal, and at times bleeding. Bleeding and a tumour in the nose point to, or at least lead to, a grave suspicion of malignant disease. In spite of the patient having some tubercular disease of the lung, he removed the inferior turbinal with Carmalt Jones' instrument. Dr. Pegler examined it microscopically, and reported that it was not malignant. Hill remarked that, looking at the specimen, many might think it was a case of intranasal lupus; but he considered the amount of inflammation present showed that the disease is rather active, and, clinically, the symptoms altogether excluded the idea that the disease was of a chronic nature, such as lupus. The patient did well, but there was a great tendency to exuberant granulations. The patient was twenty-eight years of age, married; no history of syphilis. Dr. Pegler reported that, "There appear to be several giant cell systems in the sections, but they are not typical, and the giant cells are small. The zone of leucoytes, however, is very discernible, and some of the systems are caseating in the centre. There are no bacili of tubercle to be found." It is interesting to recall Hill's concluding remarks, "Tuberculosis has been diagnosed on the microscopical appearances of the growth removed. The diagnosis was made by Dr. Pegler, in the first instance, from a view of the specimen. The latter has been seen by Mr. Bowlby, Mr. Charteris Symonds, Dr. Kanthack, and other authorities, and they have all come to the conclusion that the bulk of the growth is inflammatory, but that giant cells are present, together with breaking down nodules evidently tubercular in nature. These appearances do not support the idea that it is sarcoma or any form of malignant disease, but I think they very definitely—even though the bacillus has not been demonstrated—support the view that the disease must be tuberculous, though only of a moderately acute nature." We would ask wherein lies the pathological distinction between this tuberculous growth and lupus, unless Dr. Hill denies the identity of the specific micro-organisms of lupus and tuberculosis.

H. S. Purdon[8] records a case of ulceration of the septum nasi of a lupoid character which took place in an interesting manner. Miss D., aged twenty-four, some three years previously, nursed a favourite sister through a fatal illness due to phthisis. She frequently lent or gave her pocket-handkerchief to her sister to expectorate into. On one occasion she suffered from an ordinary "cold in the head" followed by a slight abrasion of the mucous membrane, and she attributed her disease to using the same handkerchief to "blow her nose with" on various occasions, after having been used by her consumptive sister.

**Sarcoma and Tuberculous Disease of Nasal Passages.**—Boylan[9] is able to report a successful removal of a spindle-celled sarcomatous growth from the inferior turbinated body. The patient had suffered from obstinate epistaxis, stoppage of the nose, and occasional acute pain, for several months. There was a noticeable bulging under the left nasal bone. Upon tilting up the tip of the nose a brown-red mass at once became visible, filling in the nasal passage, which was found to be united behind by the posterior naves. The growth, a solitary, soft, liver-coloured tumour, as large as a hen's egg, was removed with a wire écraseur, and the base curetted. Twenty-two months afterwards the patient announces that he finds himself with symptoms of recurrence and in excellent health. Twenty-one cases, collected from literature since Bosworth's tabulation of 1889, are referred to.

**Reflex Neuroses.**—Very numerous is the list of neuroses said to be caused reflexly by pathological conditions of the upper air passages. One fairly clear case is that reported by M. M. Boulay,[10] of Paris. A boy, aged twelve, who had suffered for two years from nocturnal epileptiform crises, with the following characteristics: Sudden awakening with anxiety, tingling of tongue, loss of consciousness, and convulsions of tongue, lips, face, and often of the four limbs, with embarrassed respiration and threatened asphyxia, the whole attack lasting five to ten minutes. The child had immense tonsils and adenoids. From the day on which the tonsils were removed the attacks ceased and never returned; the adenoids were removed later.

A case of asthma due to a foreign body in the nose is recorded by Zwillinger[11] at the Hungarian Otological and Laryngological Society. A male, aged 57, had had his nose plugged for epistaxis eight months before he came to Zwillinger, with the plug still in his nose. The left nostril was full of pus, and the fætor brain very great, while in addition there was difficulty in respiration, which became normal after the plug was removed.

G. Laurens[12] reports two cases of ocular troubles secondary to nasal disease. In the first, a male, aged 31, intranasal operation for the removal of synechiæ between the inferior turbinated bone and the septum gave sudden and permanent relief to blepharospasm of some months' duration. In the second case, a girl, aged six, left convergent strabismus disappeared a few days after operation for the removal of adenoids.

**Ozœna.**—Belfanti and Della Vedova,[13] in a communication to the Royal Society of Turin, claim that ozœna is caused by an attenuated type of diphtheria bacillus, and not by the bacillus mucosus ozœnæ. They treated a number of cases with anti-diphtheritic serum, and in half the cases the fœtor disappeared, and turgescence of mucous membrane and a fluid consistency of nasal secretions resulted. But many injections are necessary—in one case it was repeated thirty times. Professor Bozzolo, in the discussion which followed, stated that benefit had resulted in two of his cases from this treatment. Professor Gradenigo[14] has adopted the anti-diphtheritic serum treatment in sixteen cases, five of them having been diagnosed bacteriologically by Belfanti. All the cases were improved, but none cured; [illegible] he ascribes to the insufficiency of the dose employed. He confirms the specific elective action of the serum on the diseased nasal mucous membrane. Fage,[15] in his preparations of ozœnous mucus, has found along with Lowenberg's microbe some isolated cocci, bacilli, staphylococci, and rarely streptococci. The great preponderance of diplococci is very marked. The cocco-bacillus of ozœna is not localised; it is also found in the nasal pharynx, trachea, and conjunctival sac.

[1] Jour. of Laryng., July, 1896. [2] Ibid. [3] Trans. of Laryng. Soc., 1896. [4] Ibid. [5] Jour. of Laryng., July, 1896. [6] Ibid. [7] Jour. of Laryng., June, 1896. [8] Dublin Jour. of Med. Sc., Apr., 1896. [9] Jour. of Laryng., July, 1896. [10] Jour. of Laryng., July, 1896. [11] Monat. f. Ohrenheit, Oct., 189[illegible]; Jour. of Laryng., Apr., 1896. [12] Presse. Méd., Jan. 2, 1896; Jour. of Laryng., Apr., 1896. [13] Gazz. Med. di Torino, Apr. 22, 1896; Jour. of Laryng., June, 1896. [14] Arch. Ital. di Otol., vol. iv., No. 2, 1896; Jour. of Laryng. June, 1896. [15] Rev. de Laryng., Oct. 1, 1895; Jour. of Laryng., June, 1896.

